# Care coordination among pediatricians and dentists: a cross-sectional study of opinions of North Carolina dentists

**DOI:** 10.1186/1472-6831-14-33

**Published:** 2014-04-07

**Authors:** Rocio B Quinonez, Ashley M Kranz, Marshall Long, R Gary Rozier

**Affiliations:** 1 Department of Pediatric Dentistry and Pediatrics, Schools of Medicine and Dentistry, University of North Carolina at Chapel Hill, Chapel Hill, NC, USA; 2 Department of Dental Research, School of Dentistry, University of North Carolina at Chapel Hill, Chapel Hill, NC, USA; 3 Private Practice, Charlotte, NC, USA; 4 Department of Health Policy and Management, Gillings School of Global Public Health, University of North Carolina at Chapel Hill, Chapel Hill, NC, USA

**Keywords:** Care coordination, Guidelines, Infant oral health care, Dental workforce, Early childhood caries, Preventive dental services

## Abstract

**Background:**

Care coordination between physicians and dentists remains a challenge. This study of dentists providing pediatric dental care examined their opinions about physicians’ role in oral health and identified factors associated with these opinions.

**Methods:**

North Carolina general and pediatric dentists were surveyed on their opinions of how physicians should proceed after caries risk assessment and evaluation of an 18-month-old, low risk child. We estimated two multinomial logistic regression models to examine dentists’ responses to the scenario under the circumstances of an adequate and a limited dental workforce.

**Results:**

Among 376 dentists, 52% of dentists indicated physicians should immediately refer this child to a dental home with an adequate dental workforce. With a limited workforce, 34% recommended immediate referral. Regression analysis indicated that with an adequate workforce guideline awareness was associated with a significantly lower relative risk of dentists’ recommending the child remain in the medical home than immediate referral.

**Conclusions:**

Dentists’ opinions and professional guidelines on how physicians should promote early childhood oral health differ and warrant strategies to address such inconsistencies. Without consistent guidelines and their application, there is a missed opportunity to influence provider opinions to improve access to dental care.

## Background

Care coordination between physicians and dentists has been examined across the lifespan and in multiple countries [1-5]. In pediatrics, physician referrals offer an opportunity for timely care of young children and establishment of a dental home, a place where comprehensive, continuous, coordinated and family-centered cared can be delivered [6]. In the United States, the American Academy of Pediatric Dentistry (AAPD) and the American Academy of Pediatrics (AAP) recommend an oral health risk assessment and establishment of a dental home by a child’s first birthday [7-10]. In areas with limited access to dentists, the AAP recommends children at low risk for dental disease receive preventive oral health services in the medical home until a dental referral is possible [7,8]. While these guidelines continue to be updated, the fundamental recommendations on when children have dental visits have remained unchanged since 2003.

Studies indicate that physicians value oral health, believe they play an important role in preventing dental disease and promoting oral health, and report a willingness to examine children for dental disease and educate caregivers about early childhood caries [11,12]. More than 40 states reimburse physicians to deliver preventive oral health services in the first few years of life to Medicaid enrolled children; a public insurance program in the United States targeting low income families [13]. Oral health training for physicians seeking to provide fluoride varnish application varies widely by state, ranging from no training requirements in 8 states to a mandatory 90 minute continuing medical education course in North Carolina (NC) [14]. Despite widespread implementation, promoting care coordination and successful dental referrals by physicians continue to be met with difficulty [11,15-17].

Many factors can influence interprofessional collaboration and communication, ranging from personal factors such as values, expectations, attitudes, and perceptions, to broader issues including historical inter- and intra-professional conflict, varying levels of preparation, fear of diluting professional identity, complexity of care, and differences in terminology [15,18-20]. Additionally, dentists and physicians may report different barriers to care coordination. Specific to the oral health care of young children, the unwillingness of some general dentists to accept referrals from physicians may include their own perceived lack of appropriate training, discomfort in providing dental care for young children, and low reimbursement; all of which can lead to a smaller workforce available to young children [21-24]. For young children enrolled in Medicaid, an additional barrier to care is that of dentists choosing not to accept Medicaid [25].

Little is known about dentists’ opinions of the role physicians should play in providing preventive oral health services and how the opinions of dentists might influence care coordination, particularly with a limited dental workforce [26]. With more physicians providing oral health services, it is important to examine dentists’ opinions about the role of physicians in promoting oral health and the extent to which dentists agree with the AAP oral health policy statement [8]. Such information can provide insights into the challenges physicians face in delivering care according to their professional guidelines, particularly those that require active support and involvement of dentists. Additionally, it is important to examine how dentists’ opinions differ based on the availability of dentists, particularly as oral health visits in medical offices become more common than dentist visits for children aged 0–2 years in states like North Carolina that have dental workforce shortages [27,28].

This study examined NC pediatric and general dentists’ opinions about how physicians should promote oral health and identified factors associated with these opinions. Specifically, dentists provided opinions about when physicians should refer a low-risk, 18-month-old child to a dentist, what a physician should do about oral health if they thought the child should not be referred, and how these opinions would be affected by availability of dentists within their communities. The intent of using this low-risk clinical case was to present a patient with limited needs and the greatest likelihood of acceptance to both general dentists (GD) and pediatric dentists (PD). This case without treatment needs provided the ability in the analysis to assess multiple factors that might influence acceptance of patient referrals from the medical home without being dominated by the one determinant of need.

## Methods

This cross-sectional study surveyed GD and PD in NC to determine characteristics of dentists associated with opinions regarding the AAP oral health guidelines. This study was approved by the Institutional Review Board of the University of North Carolina at Chapel Hill.

### Sample

We randomly selected 1000 GD (one-third of all GD) and all PD (N = 153) currently practicing in NC from lists of licensed dentists maintained by the State Board of Dental Examiners and NC Academy of Pediatric Dentistry, respectively. Inclusion criteria for completion of the questionnaire were as follows: (1) practice of clinical dentistry in private practice >10 hours per week; (2) no current or previous participation in a postdoctoral residency program, with the exception of general practice residency, advanced education in general dentistry or postdoctoral programs in pediatric dentistry; (3) acceptance of children <12 years of age in their practice, and; (4) providing infant and toddler oral health in clinical practice. For this analysis, we applied the additional criterion that the responding dentist had to report seeing infants and toddlers in his or her practice because we are interested in improving care coordination between physicians and dentists seeing young children.

### Survey design

The 5 page survey instrument, as previously described in Long et al. [29], had 63 items, including 4 case scenarios and additional questions based on the framework assessing lack of guideline adoption in clinical practice [29,30]. This study reports results from one of the four case scenarios.

### Procedures

The survey was pilot tested by 10 dentists and subsequently mailed using the Dillman Total Design Survey Methodology [31]. Questionnaires were coded numerically and a postage-paid, preaddressed envelope was included for return. The first mailing occurred in November 2010, with a post card reminder to all participants within 2 weeks of the initial mailing. Up to 3 mailings that included a letter and questionnaire were sent to non-responders. Data collection was completed by March 2011.

### Variable construction

The dependent variable was based on responses to a case scenario asking how pediatricians should proceed after conducting a caries risk assessment and evaluation on an 18-month-old child with no dental caries, other oral pathology, behavioral or clinical risk factors. This case was selected based on findings by Long et al. [29] indicating that GD are more likely to accept a low-risk, caries-free child than a high-risk child with clinical signs of dental caries [29]. Dentists could respond with one of the following: (1) Refer the child to a dentist now; (2) Wait and refer the child at 3 years of age, but continue dental screenings during well-child visits; (3) Wait and refer the child at 3 years of age, but provide counseling and fluoride varnish during medical visits; (4) Not sure; (5) Other. Dentists were asked to respond to the case under the assumptions of an adequate and a limited dental workforce [8]. Workforce supply was intended to follow the American Academy of Pediatrics definition of availability of a dentist to accept a patient referral; however, this definition was self-defined by the survey respondent. We excluded dentists with responses of *not sure* or *other* and constructed a three group categorical dependent variable based on the first three options (reference group: refer to a dentist now).

We included survey items theorized to influence dentists’ opinions about how physicians should promote early childhood oral health. At the individual-level, we constructed binary variables indicating the dentist’s gender (reference group: female) and year of graduation from dental school during 2001–2009 (reference group: before 2001). At the practice-level, we constructed a binary variable to indicate ≥10% of the dentists’ patients were insured by Medicaid. Additionally, we constructed a categorical variable to indicate the age at which responding dentists will see a child for a first visit (1 year (reference), 2 years, 3 years or older).

Two binary variables measured awareness of the 2008 AAP oral health policy statement and 2010 AAPD infant oral health guideline [8,9]. In addition, we used eight items from a survey instrument originally developed by Tunis et al. and commonly included in questionnaires to assess providers’ attitudes about practice guidelines in general [32,33]. Overall support for guidelines was measured as the mean of responses (range = 1-4.75; mean = 2.28; Cronbach’s alpha = 0.74) to these eight questions with 5-level Likert scale type responses with the higher response indicating greater support.

To examine dentists’ support for physicians’ role in promoting oral health, we included two survey items that asked whether dental referrals by physicians are effective in increasing the percentage of infants with a dental home and whether caries-risk assessment, counseling and varnish provided by physicians reduce disease. Additionally, to assess dentists’ opinions about early childhood oral health care, we asked whether: an age one dental visit is effective in prevention of early childhood caries; whether they have to make significant changes in their schedule to incorporate infant oral health; and whether parents see the importance in dental referrals from their primary care providers. All five questions used 1–5 Likert-type response scales and each was transformed to a binary variable indicating *strongly agree* or *agree* with the statement (reference: *unsure, disagree, strongly disagree*).

### Analytical approach

Descriptive statistics were calculated for the scenario and all variables overall and by provider type. Using chi-squared tests to compare proportions and t-tests to compare means, we examined unadjusted differences in variables for GD and PD. We estimated two multinomial logistic regression models, controlling for the aforementioned variables (identified in Table 1) and using robust standard errors, to examine dentists’ responses to the scenario under the circumstances of an adequate and a limited dental workforce [34]. Regression models included both GD and PD because of the limited sample size of PDs, their limited variability in responses to the case scenario, and their limited variability in responses to many explanatory variables. Relative risk ratios (RR) were calculated to indicate the relative risk of a dentist responding with wait and refer the child at age 3 rather than adhering to AAPD guidelines (i.e., immediate referral). Z-tests and 95 percent confident intervals (CI) were used to examine the association between independent variables and scenario responses. All tests were performed in Stata/IC 12.1 (Statacorp, College Station, TX) using a 0.05 significance level [34].

**Table 1:** Characteristics of dentists included in the analytical sample

**Mean (standard deviation) or %**	**All (N = 379)**	**General dentists who treat infants and toddlers (N = 279)**	**Pediatric dentists (N = 100)**
***Individual characteristics***			
Pediatric dentist	26.4	0	100***
Graduated dental school before 2001	29.5	32.6	20.6*
Male	64.9	68.8	54**
***Practice characteristics***			
10% or more of patients in practice are Medicaid-insured	50.7	40.5	79***
Age dentist will see child for first visit			
1 year	55.7	45.2	85***
2 years	11.6	15.4	1***
3 years or older	26.1	34.1	4***
***Awareness of guidelines***			
Aware of 2008 AAP infant oral health guidelines [8]	52.9	39.9	89***
Aware of 2010 AAPD infant oral health guidelines [9]	55.9	40.2	99***
***Opinion of guidelines***			
Scale measuring opinions about guidelines	2.3 (0.5)	2.4 (0.5)	2.1 (0.4)***
***Agreement about physicians role in promoting oral health***			
Dental referrals by physicians are effective in increasing the percentage of infants with a dental home^a^	78.1	73.8	90***
Caries risk assessment, counseling & varnish provided by physicians decreases disease in infants and toddlers^a^	76.8	78.5	72
***Opinions about infant and toddler oral health care***			
Age one dental visit is effective in prevention of early childhood caries^a^	71.0	64.5	89
Have to make significant changes in my schedule to incorporate infant oral health care^a^	17.2	21.5	5***
Parents see the importance in dental referrals from physicians	59.4	55.6	70*

P-values from t-test for continuous variables and chi-squared test for binary and categorical variables. *p < 0.05, **p < 0.01, ***p < 0.001.

Sample size varies for a few variables listed above: graduation year (n = 376); percent Medicaid patients (n = 367); referrals from; age dentist will see for first visit (n = 354); aware of AAP guidelines (n = 378); aware of AAPD guidelines (n = 376); opinions about guidelines (n = 371).

^a^Indicates response of *strongly agree* or *agree* (reference: *unsure, disagree, strongly disagree*).

## Results

Of 1000 questionnaires mailed, 596 were returned for a response rate of 59.6%. We excluded 73 respondents who practiced clinical dentistry <10 hours per week and 144 who do not treat children <12 years. An additional 135 GDs were excluded because they did not treat infants and toddlers. Of the 379 remaining dentists, complete case analysis was used to examine predictors of how dentists think physicians should refer with adequate (n = 305) and limited dental workforce (n = 303).

Unadjusted analyses indicate that PD and GD differed on nearly all measures examined (Table 1). PD had more Medicaid patients, were more likely to see 1 year olds, and were more likely to be aware of infant oral health guidelines. Although PD and GD agreed that caries-risk assessment, counseling and fluoride varnish provided by physicians can decrease disease in early childhood, PD were more likely to agree that referral by physicians increase dental homes.

In response to the case scenario and consistent with AAPD guidelines, 52% of dentists recommended that pediatricians immediately refer the child to a dentist when adequate dental workforce existed, compared to 34% in an environment with limited dental workforce (Table 2). In a limited dental workforce environment, 26% of dentists recommended pediatricians wait and refer at 3 years of age, but provide counseling and fluoride varnish during medical visits, in line with AAP guidelines. Responses to this scenario differed for PD and GD. In both workforce situations, PD were more likely to recommend immediate referral.

**Table 2:** Distribution of dentists' responses to case scenario

**Case description.** An 18 month old child receives a caries risk assessment and screening by a pediatrician and was found to have no dental decay or oral pathology. No behavioral or clinical caries risk factors are reported. Based on your determination of the child's risk assessment above, how should the pediatrician address the child's oral health needs?	**All (N = 376)**		**General dentists who treat infants and toddlers (N = 276)**		**Pediatric dentists (N = 100)**	
**Adequate workforce**	**N**	**%**	**N**	**%**	**N**	**%**
Refer the child to a dentist now	195	51.9	111	40.2	84	84
Wait and refer the child at 3 years of age, but continue dental screenings during well-child visits	121	32.2	115	41.7	6	6
Wait and refer the child at 3 years of age, but provide counseling and fluoride varnish during medical visits	49	13	42	15.2	7	7
Not sure	1	0.3	1	0.4	0	0
Other	10	2.6	7	2.5	3	3
**Limited workforce**						
Refer the child to a dentist now	127	33.8	74	26.1	55	55
Wait and refer the child at 3 years of age, but continue dental screenings during well-child visits	139	36.9	121	43.8	18	18
Wait and refer the child at 3 years of age, but provide counseling and fluoride varnish during medical visits	97	25.8	73	26.5	24	24
Not sure	3	0.8	2	0.7	1	1
Other	10	2.7	8	2.9	2	2

The results of chi-squared tests indicate that dentists’ response to the 3-group categorical case scenario differed significantly for general and pediatric dentists. (adequate workforce: *χ*^2^ = 60.0, p < 0.000; limited workforce: *χ*^2^ = 31.5 p < 0.000).

The results of the regression models presented in Table 3 indicate the relative risk of a dentist responding with wait and refer the child at age 3 (with or without providing varnish) rather than adhering to AAPD guidelines (i.e., immediate referral). With an adequate dental workforce, dentists who were recently trained, male, agreed that oral health services provided by physicians decrease disease, and believed that parents see the importance in referrals from physicians had a significantly higher relative risk of recommending physicians wait to refer and provide varnish in the medical office relative to immediate referral. Dentists who see children for their first visit at age 2 years or older as compared to 1 year had significantly higher relative risk of recommending physicians wait to refer the child. Dentists aware of the AAP guidelines had a significantly lower relative risk of recommending physicians wait and provide varnish (RR = 0.25, 95% CI = 0.08, 0.78), whereas dentists aware of the AAPD guidelines had a significantly lower relative risk of recommending that physicians wait to refer without varnish (RR = 0.27, 95% CI = 0.09, 0.84).

**Table 3:** **Predictors of dentists' referral response for a low-risk 18 month old child**^a^

	**Adequate workforce (N = 305)**		**Limited workforce (N = 303)**	
**Variables**	**Wait and refer the child at age 3, but continue screenings**	**Wait and refer the child at age 3, but provide varnish**	**Wait and refer the child at age 3, but continue screenings**	**Wait and refer the child at age 3, but provide varnish**
***Individual characteristics***				
Graduated dental school before 2001	1.84 [0.86, 3.93]^b^	2.81* [1.15, 6.85]	0.99 [0.49, 2.00]	1.82 [0.91, 3.65]
Male	1.75 [0.83, 3.73]	2.91* [1.08, 7.84]	1.08 [0.54, 2.18]	1.40 [0.7, 2.9]
***Practice characteristics***				
≥10% patients in practice are Medicaid-insured	0.89 [0.44, 1.78]	1.46 [0.65, 3.27]	0.84 [0.44, 1.61]	1.38 [0.71, 2.67]
Age dentist will see child for first visit (ref: 1 year)				
2 years	3.94** [1.42, 10.90]	4.51* [1.33, 15.32]	1.90 [0.7, 5.2]	1.72 [0.58, 5.07]
3 years or older	5.21*** [2.18, 12.48]	4.46** [1.71, 11.64]	2.67* [1.13, 6.31]	2.01 [0.86, 4.72]
***Awareness of guidelines***				
Aware of 2008 AAP infant oral health guidelines	1.32 [0.45, 3.90]	0.25* [0.08, 0.78]	1.49 [0.51, 4.33]	0.74 [0.30, 1.86]
Aware of 2010 AAPD infant oral health guidelines	0.27* [0.09, 0.84]	1.06 [0.37, 3.06]	0.30 [0.1, 1.0]	1.00 [0.4, 2.7]
***Opinion of guidelines***				
Scale measuring opinions about guidelines	1.80 [0.9, 3.6]	1.05 [0.46, 2.42]	1.98 [0.96, 4.09]	1.43 [0.68, 3.02]
***Agreement about physicians role in promoting oral health***				
Dental referrals by physicians are effective in increasing % of infants with a dental home	0.38* [0.15, 0.95]	0.24* [0.07, 0.79]	0.38* [0.16, 0.90]	0.30* [0.11, 0.77]
Caries risk assessment, counseling & varnish provided by physicians decreases disease in infants and toddlers	1.51 [0.65, 3.53]	4.70* [1.2, 18.3]	1.02 [0.48, 2.16]	3.70** [1.5, 9.5]
***Opinions about infant and toddler oral health care***				
Age 1 dental visit is effective in ECC prevention	0.28***[0.13, 0.58]	0.24** [0.10, 0.62]	0.43* [0.20, 0.91]	0.60 [0.3, 1.4]
Have to make significant changes in my schedule to incorporate infant oral health care	1.57 [0.60, 4.11]	0.44 [0.11, 1.78]	1.20 [0.53, 2.73]	0.46 [0.15, 1.41]
Parents see the importance in dental referrals from physicians	1.60 [0.78, 3.28]	3.49* [1.23, 9.89]	1.77 [0.93, 3.38]	2.92** [1.39, 6.12]

^a^Reference group: Refer to a dentist now; ^b^Relative risk ratios followed by 95% confidence intervals in brackets. *p < 0.05, **p < 0.01, ***p < 0.001.

With a limited dental workforce, dentists who agree that referrals by physicians promote dental homes had a significantly lower relative risk of recommending physicians should wait to refer the child (RR = 0.38, 95% CI = 0.16, 0.90) or wait to refer while providing varnish (RR = 0.30, 95% CI = 0.11, 0.77) relative to immediate referral. Dentists who thought the age one dental visit is effective in preventing ECC had a significantly lower relative risk of recommending physicians wait to refer without varnish (RR = 0.43, 95% CI = 0.20, 0.91). Relative to immediate referral, dentists had a significantly higher relative risk of recommending physicians wait to refer and provide varnish in the medical office if they agreed that oral health services provided by physicians decrease disease (RR = 3.70, 95% CI = 1.45, 9.45) or had the opinion that parents see the importance in dental referrals from physicians had a (RR = 2.92, 95% CI = 1.39, 6.12).

## Discussion

This study of NC dentists who see infants and toddlers examined dentists’ opinions about how physicians should promote oral health in early childhood. Using a case scenario of an 18-month-old low-caries-risk child provided an optimal situation whereby dentists would be most likely to provide care because no restorative treatment was needed. Our findings highlight the variation in dentists’ opinions about physicians’ role in preventing dental disease, and the factors influencing dentists’ opinions, including guideline awareness and dental workforce availability.

We observed that almost one-half of dentists recommended physicians refer a low-risk 18 month old to a dental home at 3 years of age regardless of dentist availability, which differs from the AAPD recommendations of referring all children at one year. Responses to the case scenario varied by provider type, with 84% and 40% of PD and GD, respectively supporting referral now. A prior study reported similar findings, but did not examine the role of workforce availability in dentists’ opinions regarding the age 1 dental visit [22].

When faced with a limited dental workforce, 34% of dentists recommended immediate referral, 37% recommended wait and refer with continued screenings during well-child visits, and 26% agreed that physicians should provide preventive oral health services including fluoride varnish during medical visits. No difference between PD and GD existed with the latter. The majority of dentists believed that a low-risk child should not receive professional-applied fluoride in medical homes. It is uncertain whether provider responses indicate opposition to physicians delivering oral health services or confusion due to varying guidelines. The most recent AAP policy statement (2008) states that administration of fluoride varnish by medical practitioners is appropriate for patients with significant risk, but also recommends annual once yearly fluoride application for low risk children [8]. This recommendation differs from AAPD dental guidelines stating that children with low caries risk may not receive additional benefit from topical fluoride application [35]. Additionally, we observed varying responses to how a low risk child should be triaged based on dental workforce availability. Thus, organizations should consider promoting uniform guidelines across professions that better reflect the current availability of dentists to encourage care coordination [36,37].

Regression results indicate that dentists were more likely to follow AAPD guidelines for the low-risk child in the case scenario if they agreed that referrals increase dental homes, believed the age one dental visit prevents ECC, and were aware of guidelines [23]. While positive infant oral health attitudes, including guideline awareness, continue to be an important component influencing provider practice behaviors, strategies should focus on referral environments because promoting referrals may be even more important, above provider opinions, in promoting coordinated care [20,22,38,39]. Early referral to a dental home, however, has been historically met with some reservation from dentists [40]. Dentists report parents’ lack of value for the age 1 visit as the most common barrier to performing infant evaluations, thus highlighting the importance of targeting parents to encourage successful referrals [41].

Strategies aimed at promoting coordinated care should support providers already collaborating and also encourage those who are not yet engaged. To encourage GDs to see young children, Garg and colleagues identified training and the presence or access to a pediatric dentist consultant as desired and potential facilitators [21]. Engaging educational interventions for primary care providers, like small group discussions and interactive workshops, may also help promote behavior change [42]. For example, Chapter Oral Health Advocates (COHA) trained physicians from 64 AAP Chapters to collaborate with physicians and dentists in their states to increase oral health awareness [43]. Similarly, the Carolina Dental Home Initiative aims to fortify relationships between physicians and dentist to facilitate and improve referrals to dental homes [44]. These types of initiatives require further assessment to determine their long term impact on providers’ opinions and oral health outcomes.

Medical and dental school education provides an opportunity to promote interprofessional collaboration and encourage positive referral environments; particularly in light of our findings that recent graduates were less adherent to the AAPD guidelines than older graduates. New modifications in accreditation standards for dental education programs now incorporate collaboration with health care professionals and a standard of competency in communicating and collaborating with other members of the health care team to facilitate the provision of health care [45]. Educational strategies on how best to promote collaborative care require further attention. A study by Chung and colleagues examined dental and medical students’ knowledge and opinions on infant oral health [46]. While dental students were more likely than medical students to provide correct responses regarding the timing and importance of the age 1 dental visit, fourth year dental students were less likely than first year dental students to recommend and agree it is important to establish a dental home by age 1 [46]. While counterintuitive, the authors concluded that increased experience may not always lead to desired results. Consideration of how increased student debt may influence their desire to provide less financially rewarding services warrants consideration, as well as the role of gender in promoting positive opinions regarding physician referral.

This study should be considered in the context of its limitations. First, we examined only dentists who care for infants and toddlers in NC. Because few NC dentists see infants and toddlers, we were unable to estimate separate regression models for PDs and GDs or examine interaction effects among variables. Strategies to engage dentists who do not currently see infants and toddlers may need to be different. Second, although case scenarios are widely used in simulating clinical practice, our specific case did not specify insurance type or financial status of the patient, possibly influencing responses. Third, although participation was lower than desired at 59%, it is consistent or better than other survey responses in this area [39,41,47]. Finally, the generalizability of these findings is in the context of a state that has been engaged in the collaboration of physician involvement for more than a decade. Opinions may vary in other states and countries based on the level of established collaborations.

## Conclusions

This study is the first to examine dentists’ opinions regarding early childhood oral health promotion in medical homes. To reach children early and help ameliorate the negative sequelae associated with dental disease, collaboration between medicine and dentistry will continue to be critical, as more physicians deliver preventive oral health services [7,8,12,48,49]. While this study did not comprehensively examine all of the important influences that impact child oral health, it highlights areas that can be addressed to improve care coordination [50]. Strategies to increase guideline awareness and bring greater consistency between professional organizations should be examined and evaluated for their impact on the oral health of infants and toddlers. Education during and following professional training can provide an opportunity to help shape opinions and promote collaborative models of care.

## Abbreviations

AAPD: American academy of pediatric dentistry; AAP: American academy of pediatrics; NC: North Carolina; GD: General dentists; PD: Pediatric dentists; CI: Confidence interval; RR: Relative risk.

## Competing interests

The authors declare that they have no competing interest.

## Authors’ contributions

RQ: conceptual design, data acquisition, analysis, manuscript preparation. AK: conceptual design, analysis, manuscript preparation. ML: conceptual design, data acquisition. GR: conceptual design, data acquisition, analysis, manuscript preparation. All authors read and approved the final manuscript.

## Pre-publication history

The pre-publication history for this paper can be accessed here:

http://www.biomedcentral.com/1472-6831/14/33/prepub
